# Incidence and Genomic Background of Antibiotic Resistance in Food-Borne and Clinical Isolates of *Salmonella enterica* Serovar Derby from Spain

**DOI:** 10.3390/antibiotics12071204

**Published:** 2023-07-19

**Authors:** Xenia Vázquez, Raquel García-Fierro, Javier Fernández, Margarita Bances, Ana Herrero-Fresno, John E. Olsen, Rosaura Rodicio, Víctor Ladero, Vanesa García, M. Rosario Rodicio

**Affiliations:** 1 Departamento de Biología Funcional, Área de Microbiología, Universidad de Oviedo (UO), 33006 Oviedo, Spain; xenia.vazquez@ipla.csic.es (X.V.); raquel.garciafierro@efsa.europa.eu (R.G.-F.); 2 Grupo de Microbiología Traslacional, Instituto de Investigación Sanitaria del Principado de Asturias (ISPA), 33011 Oviedo, Spain; javier.fernandezd@sespa.es (J.F.); mrosaura@uniovi.es (R.R.); 3 Instituto de Productos Lácteos de Asturias (IPLA), Consejo Superior de Investigaciones Científicas (CSIC), 33300 Villaviciosa, Spain; ladero@ipla.csic.es; 4 Servicio de Microbiología, Hospital Universitario Central de Asturias (HUCA), 33011 Oviedo, Spain; 5 Centro de Investigación Biomédica en Red-Enfermedades Respiratorias, 30627 Madrid, Spain; 6 Research & Innovation, Artificial Intelligence and Statistical Department, Pragmatech AI Solutions, 33001 Oviedo, Spain; 7 Laboratorio de Salud Pública, Dirección General de Salud Pública, Consejería de Salud del Principado de Asturias, 33011 Oviedo, Spain; margaritabg@educastur.org; 8 Department of Veterinary and Animal Sciences, Faculty of Health and Medical Sciences, University of Copenhagen, 1870 Frederiksberg, Denmark; ahefr@food.dtu.dk (A.H.-F.); jeo@sund.ku.dk (J.E.O.); 9 Departamento de Bioquímica y Biología Molecular, Universidad de Oviedo (UO), 33006 Oviedo, Spain; 10 Grupo de Microbiología Molecular, Instituto de Investigación Sanitaria del Principado de Asturias (IAPA), 33011 Oviedo, Spain; 11 Laboratorio de Referencia de Escherichia coli (LREC), Departamento de Microbioloxía e Parasitoloxía, Facultade de Veterinaria, Campus Terra, Universidade de Santiago de Compostela, 27002 Lugo, Spain; 12 Instituto de Investigación Sanitaria de Santiago de Compostela, 15706 Santiago de Compostela, Spain

**Keywords:** *Salmonella enterica* serovar Derby, ST40, SPI-23, antimicrobial drug resistance, *fosA7.3*, SGI1-like, resistance plasmid, IncI1-I(α), pSC101-like, phylogenetic analysis

## Abstract

*Salmonella enterica* serovar Derby (*S*. Derby) ranks fifth among nontyphoidal *Salmonella* serovars causing human infections in the European Union. *S*. Derby isolates (36) collected between 2006 and 2018 in a Spanish region (Asturias) from human clinical samples (20) as well as from pig carcasses, pork- or pork and beef-derived products, or wild boar (16) were phenotypically characterized with regard to resistance, and 22 (12 derived from humans and 10 from food-related samples) were also subjected to whole genome sequence analysis. The sequenced isolates belonged to ST40, a common *S*. Derby sequence type, and were positive for SPI-23, a *Salmonella* pathogenicity island involved in adherence and invasion of the porcine jejune enterocytes. Isolates were either susceptible (30.6%), or resistant to one or more of the 19 antibiotics tested for (69.4%). Resistances to tetracycline [*tet*(A), *tet*(B) and *tet*(C)], streptomycin (*aadA2*), sulfonamides (*sul1*), nalidixic acid [*gyrA* (Asp87 to Asn)] and ampicillin (*bla*_TEM-1_-like) were detected, with frequencies ranging from 8.3% to 66.7%, and were higher in clinical than in food-borne isolates. The *fosA7.3* gene was present in all sequenced isolates. The most common phenotype was that conferred by the *tet*(A), *aadA2* and *sul1* genes, located within identical or closely related variants of *Salmonella* Genomic Island 1 (SGI1), where mercury resistance genes were also present. Diverse IncI1-I(α) plasmids belonging to distinct STs provided antibiotic [*bla*_TEM-1_, *tet*(A) and/or *tet*(B)] and heavy metal resistance genes (copper and silver), while small pSC101-like plasmids carried *tet*(C). Regardless of their location, most resistance genes were associated with genetic elements involved in DNA mobility, including a class one integron, multiple insertion sequences and several intact or truncated transposons. By phylogenetic analysis, the isolates were distributed into two distinct clades, both including food-borne and clinical isolates. One of these clades included all SGI1-like positive isolates, which were found in both kinds of samples throughout the entire period of study. Although the frequency of *S*. Derby in Asturias was very low (0.5% and 3.1% of the total clinical and food isolates of *S. enterica* recovered along the period of study), it still represents a burden to human health linked to transmission across the food chain. The information generated in the present study can support further epidemiological surveillance aimed to control this zoonotic pathogen.

## 1. Introduction

Nontyphoidal serovars (NTS) of *Salmonella enterica* subspecies *enterica* are zoonotic pathogens that constitute a leading cause of acute gastroenteritis in humans [1,2]. In the European Union (EU), NTS are the second most frequently informed zoonotic bacteria involved in human infections, and the most common cause of bacterial food-borne outbreaks [3]. In addition, NTS are potential reservoirs for antimicrobial resistance genes, which can be transferred to humans through the food chain, hence contributing to the burden of resistance in human medicine [4].

*S. enterica* subspecies *enterica* serovar Derby (*S*. Derby) is a NTS which, according to the White—Kauffmann—Le Minor scheme, has the antigenic formula 1,4,[5] [12]:f,g:[1,2], belonging to serogroup O:4 (B) [5]. In the last few years, *S*. Derby has been ranked as the fifth most common cause of human salmonellosis in the EU, with 474, 525 and 719 confirmed cases in 2021, 2020 and 2019, respectively [3]. *S*. Derby has primarily been linked to the pig sector, but it has also been detected in other food-producing animals, like poultry, cattle and wild boar [3]. The presence of *S*. Derby in food-producing animals and derived products is a cause of concern because of its possible transmission to humans through the food chain.

Like other NTS, *S*. Derby usually causes self-limiting gastroenteritis that manifests with fever, acute diarrhoea, abdominal cramps, nausea and sometimes vomiting, and typically resolves without treatment with antibiotics. However, therapy becomes necessary to combat serious infections, including bacteraemia, particularly when immunocompromised individuals, children and people of advanced age are involved. In these cases, resistance, and particularly multidrug resistance, can negatively impact the outcome of the disease.

Multiple genetic elements involved in DNA mobility, including genomic islands, plasmids, integrons, insertion sequences and transposons, play a pivotal role in acquisition and dissemination of antimicrobial drug resistance among bacteria [6]. Recombination and transposition events can lead to the accumulation of multiple resistance genes within complex regions, which may be located on the chromosome or carried by plasmids. As an example, resistance to several antibiotics in *S. enterica* can be mediated by a chromosomal genomic island integrated at the 3′-end of the *trmE* gene (also designated *thdF* or *mnmE*). This island, known as SGI1 (*Salmonella* Genomic Island 1), was first reported in the *S*. Typhimurium definitive phage type 104 (DT104) pandemic clone [7]. It consists of a 27 kb backbone, which includes the *int* gene responsible for integration and excision of the island and several genes associated with mobilization. In addition, it has a 15 kb resistance region with features of a complex class 1 integron designated In104, which is located close to the 3′-end of the island, upstream of the *yidY* gene [8,9,10]. Later, SGI1 or variants herein have also been reported in other phage types of *S*. Typhimurium and many other serovars of *S. enterica* including *S*. Derby, as well as in different bacterial species [9,10,11,12].

*S*. Derby has traditionally been typed by Pulsed-Field Gel Electrophoresis (PFGE) and other phenotypic and genotypic techniques [13,14,15,16]. These early studies, mainly performed with pig and human isolates, showed a relatively wide range of PFGE and antibiotic resistance profiles, frequently involving streptomycin, sulfonamides and/or tetracycline. However, traditional techniques have a relatively low discriminatory power, when compared with the highly discriminative approaches that arose with the advent of affordable whole genome sequencing (WGS) [17]. Recently, such approaches have been applied to investigate food, animal, environmental and human isolates of *S*. Derby from the United Kingdom (UK), France and Germany, establishing their phylogenetic relationships [17,18,19,20,21,22,23]. In the UK, two distinct lineages were identified which were apparently adapted to different food-producing animals, i.e., pigs and turkeys, and varied by the presence or absence of *Salmonella* pathogenicity island 23 (SPI-23), proposed to be involved in the invasion of porcine enterocytes [19,20,24]. A broader genomic study performed in France confirmed the polyphyletic nature of *S*. Derby, expanding the number of detected lineages to three [22,23]. Each of these lineages was associated with separate sequence types (ST39-ST40, ST71 and ST682), and was linked to a specific animal host, particularly pork (ST39-ST40 and ST682) or poultry (ST71). ST71 and ST682 isolates were mostly pan-susceptible; ST39 showed limited resistance while ST40 displayed the highest level of resistance [18,22]). The latter are primarily characterized by the streptomycin, sulfonamide and tetracycline multidrug resistant profile, encoded by the *aadA2*, *sul1* and *tet*(A) genes located on a variant of the SGI1 genomic island [22,25]. ST39, ST40 and ST682 were also associated with *S*. Derby isolates recovered across Germany in pig and cattle slaughterhouses [18]. In this country, nearly three quarters of the isolates analyzed carried at least one resistance determinant, with a higher numbers of genes found in ST40 isolates. Interestingly, the *fosA7* or *fosA7.3* genes have been reported in ST39 or ST40 *S*. Derby isolates, respectively, sequenced after the inclusion of *fosA* alleles in the ResFinder database [18,22].

No detailed information is currently available on isolates of *S*. Derby circulating in livestock and causing human infections in Spain. To overcome this limitation, in the present investigation all isolates of this serovar recovered from food-related samples and human clinical samples in a Northern Spanish region (Asturias) from 2006 to 2018 were thoroughly characterized, using both conventional and WGS approaches.

## 2. Materials and Methods

### 2.1. Bacterial Isolates

All *S*. Derby isolates registered at the “Laboratorio de Salud Pública” (LSP, Asturias, Spain), between 2006 and 2018 (*n* = 36), were included in the study (Table 1). Sixteen derived from food-related samples, specifically pig carcasses at slaughter (3), fresh pork sausages (2), pork and beef minced meat (10) and wild boar minced meat (1). Another 20 were recovered from human clinical samples including feces (19) and urine (1), each isolated from a different patient. Serotype was determined phenotypically either at the “Centro Nacional de Microbiología” (CNM, Madrid, Spain) in the case of clinical isolates or at the “Agencia Española de Consumo, Seguridad Alimentaria y Nutrición” (AECOSAN, Madrid, Spain) for food isolates.

### 2.2. Antimicrobial Susceptibility Testing and Detection of Resistance Genes

Antimicrobial susceptibility was determined by disc diffusion assays using commercial discs (Oxoid, Madrid, Spain). The following compounds, for which resistance was reported in *S. enterica*, were tested: ampicillin (10), amoxicillin-clavulanic acid (30), cefepime (30), cefotaxime (30), cefoxitin (30), ertapenem (10), amikacin (30), gentamicin (10), streptomycin (10), nalidixic acid (30), ciprofloxacin (5), kanamycin (30), tobramycin (10), azithromycin (15), chloramphenicol (30), tetracycline (30), sulfonamides (300), trimethoprim (5) and nitrofurantoin (300), with the numbers in parenthesis corresponding to the amount per disk expressed in µg. Results were interpreted according to EUCAST (European Committee on Antimicrobial Susceptibility Testing) guidelines (https://eucast.org/clinical_breakpoints/; last accessed on 8 June 2023), except in the case of nalidixic acid, for which CLSI criteria were used [26]. Isolates were regarded as multidrug resistant (MDR) when they were resistant to three or more antibiotics of different families [27]. Based on the resistance phenotypes, genes known to be involved in resistance to ampicillin (*bla*_TEM-1_-like, *bla*_OXA-1_-like, *bla*_PSE-1_), streptomycin (*strA*, *strB*, *aadA1*-like and *aadA2*), sulfonamides (*sul1*, *sul2*, *sul3*) and tetracycline [*tet*(A), *tet*(B), *tet*(C) and *tet*(G)] were screened by PCR amplification using previously reported primers and conditions [28,29,30]; see Table S1 for detailed information regarding the primers, and Figure S1 for representative examples of the obtained amplicons. Although resistance to fosfomycin was not experimentally tested, the presence of *fosA7*-like genes was screened in all isolates, also by PCR with primers designed for the present study (Table S1; Figure S1).

### 2.3. Whole Genome Sequencing and Bioinformatics Analysis

Twenty-two isolates, ten derived from food-related samples and twelve from human clinical samples, were subjected to WGS. The selection encompasses the diversity of resistance gene profiles. Genomic DNA was purified from the isolates using the GenElute Bacterial Genomic DNA Kit (Sigma-Aldrich; Merk Life Science, Madrid, Spain) according to the manufacturer’s instructions. WGS was accomplished with Illumina technology at the sequencing facilities of Eurofins Genomics (Ebersberg, Germany), BGI Genomics (Beijing, China) or the Veterinary Clinical Microbiology section of the Department of Veterinary and Animal Science (University of Copenhagen, Denmark). Paired-end reads of 150 nt were obtained from approximately 400–500 bp libraries and assembled de novo with Spades v3.14.0 [31]. The resulting contigs were submitted to the GenBank database and annotated by the NCBI Prokaryotic Genome Annotation Pipeline (PGAP) [32]. Information related to the quality of the assemblies is compiled in Table S2.

Bioinformatics analysis was performed with the SeqSero (version 1.2), MLST (version 2.0), ResFinder (version 4.1), PlasmidFinder (version 2.1) and pMLST (version 2.0) tools, available at the Center for Genomic Epidemiology (CGE) of the Technical University of Denmark (DTU) (https://www.genomicepidemiology.org/; last accessed on 6 June 2023). Plasmid profiles of the isolates harboring plasmid-located resistance genes are shown in Figure S2. To establish the genetic environment of resistance genes, the annotation of relevant contigs was manually refined and further assemblies were achieved with the aid of BLAST (https://blast.ncbi.nlm.nih.gov; last accessed on 16 June 2023) and Clone Manager 9. When required, PCR reactions were performed to confirm the correct assemblies (primers available upon request). Graphic representations of the resistance regions were performed with Easyfig v2.1 [33]. SPIs were screened by using SPIFinder (version 2.0; CGE, DTU) and VFDB (Virulence Factor DataBase; http://www.mgc.ac.cn/VFs/search_VFs.htm; last accessed 6 June 2023), and the information was complemented with a customized database searched with MyDbFinder (version 2.0; CGE, DTU).

### 2.4. Phylogenetic Analysis

The phylogenetic relationships between the sequenced isolates were inferred by using the CSI phylogeny tool (version 1.4; CGE, DTU). The pipeline was run with default parameters, using the genome of LSP 138/08 as the reference for SNP calling. For bootstrap support of the consensus tree, 1000 replicates were generated [34]. The resulting SNP matrix is shown in Table S3.

## 3. Results

### 3.1. Origin of the Isolates and General Features of the Sequenced Genomes

Between 2006 and 2018, a total of 36 isolates of *S*. Derby were detected in Asturias. They were recovered from food samples (16) or human clinical samples (20). As indicated before, all these isolates were phenotypically characterized for resistance, and 22 of them (10 from food samples and 12 from clinical samples) were sequenced with short-read Illumina technology (Table 1 and Table 2). While each of the human isolates derived from a different patient, certain food-borne isolates were recovered from the same sample, i.e., pig carcasses (LSP 32/15 and LSP 33/15) or pork and beef minced meat (LSP 82/16 and LSP 83/16 or LSP 101/16, LSP 102/16 and LSP 103/16). Further characterization supported that the isolates from the same sample belonged to the same strain (Table 1). In this way, only 12 food-related strains were identified.

The assembled size of the genomes ranged between 4.76 and 5.08 Mb, with a GC content of approximately 52%. All sequenced isolates were confirmed as *S*. Derby by means of SeqSero (4:f,g:-), and assigned to ST40 by MLST. Plasmid replicons were detected in 15 of the sequenced isolates, in numbers ranging from 1 up to 3. They were recognized as IncI1-I(α), IncQ1, IncY, IncP, p0111 or Col (pHAD28) by PlasmidFinder, while pSC101-like plasmids were also detected by BLASTn comparisons of relevant contigs. Plasmids containing resistance genes were selected for further characterization (Section 3.3.2). SPI-1 to SPI-6, SPI-9 and SPI-23 pathogenicity islands, as well as incomplete versions of SPI-11 and SPI-13, were found in all isolates sequenced, whilst part of SPI-12 was present in nearly half of them (10 out of 22; 45.5%).

### 3.2. Resistance Phenotypes and Genetic Bases of the Observed Resistances

According to disk diffusion assays, 11 isolates (30.6%) were susceptible to all antimicrobials tested, while 25 (69.4%) were resistant to 1 or more compounds (Table 1). Resistances to tetracycline, sulfonamides, streptomycin, nalidixic acid and ampicillin were shown by 24 (66.7%), 17 (47.2%), 17 (47.2%), 6 (16.7%) and 3 (8.3%) isolates, respectively. The most frequent combination included resistances to streptomycin, sulfonamides and tetracycline, which appeared either alone (13 isolates; 36.1%) or together with additional resistances to ampicillin or nalidixic acid (1 and 3 isolates, respectively) (Table 1). Overall, 18 isolates (50%) were MDR, as they were resistant to 3 or more antibiotics belonging to different families.

Based on PCR detection, *tet*(A), *tet*(B) and *tet*(C) tetracycline resistance genes were present in 18, 2, and 4 isolates, respectively. All streptomycin, sulfonamide and ampicillin resistant isolates yielded the amplicons expected for *aadA2* (17), *sul1* (17) and *bla*_TEM-1_ (3), respectively. The same point mutation in the *gyrA* gene, i.e., GAC to AAC that changed Asp87 into Asn in the protein, was detected by WGS in four out of the six nalidixic acid resistant isolates. In the remaining two, the genetic bases of this resistance could not be established. By combining the resistance phenotypes with responsible genes, a total of 10 profiles were identified, which were termed as “R0 (for susceptible isolates) to R9” (Table 1). The most common profile was R6, which comprised resistances to sulfonamides, streptomycin and tetracycline, encoded by the *aadA2*, *sul1* and *tet*(A) genes. Resistance to fosfomycin was not tested for, but the *fosA7.3* gene variant was found in all sequenced genomes (Section 3.3.1), and a PCR screening revealed the presence of a *fosA7*-like gene in all isolates. The *acc(6)-1aa* gene and the Thr57Ser substitution in the ParC protein were also detected in all sequenced isolates, but they were not associated with aminoglycoside or quinolone resistance.

### 3.3. Genetic Environment of the Resistance Genes

**Figure 1:**
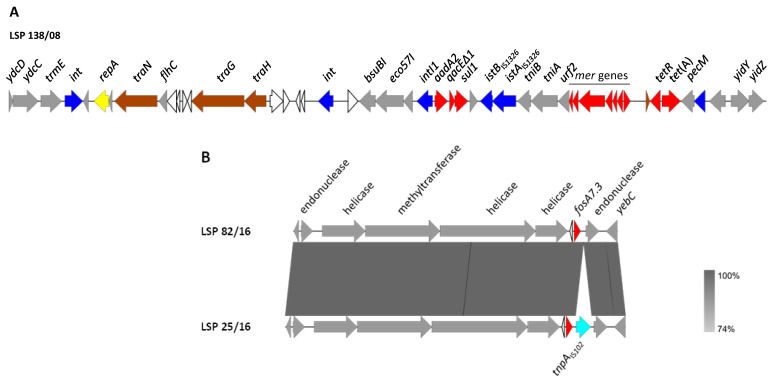
Genetic environment of chromosomal resistance genes found in *S*. Derby isolated from human clinical samples and food samples in Spain. (**A**). Genetic organization of the SGI1-variant, using LSP 138/08 as a model. (**B**). Comparison of the genetic context of *fosA7.3* in LSP 82/16 (used as a model) and LSP 25/16. The alignments were created with Easyfig BLASTn. The gray shading between regions reflects nucleotide sequence identities according to the scale shown at the right lower corner of the figure. Genes are represented by arrows pointing to the direction of transcription. Genes with similar functions are shown in the same color. Color code: yellow, DNA replication; brown, conjugal transfer; blue, genes involved in DNA mobility; red, resistance genes; grey, other functions; white, hypothetical proteins.

**Figure 2:**
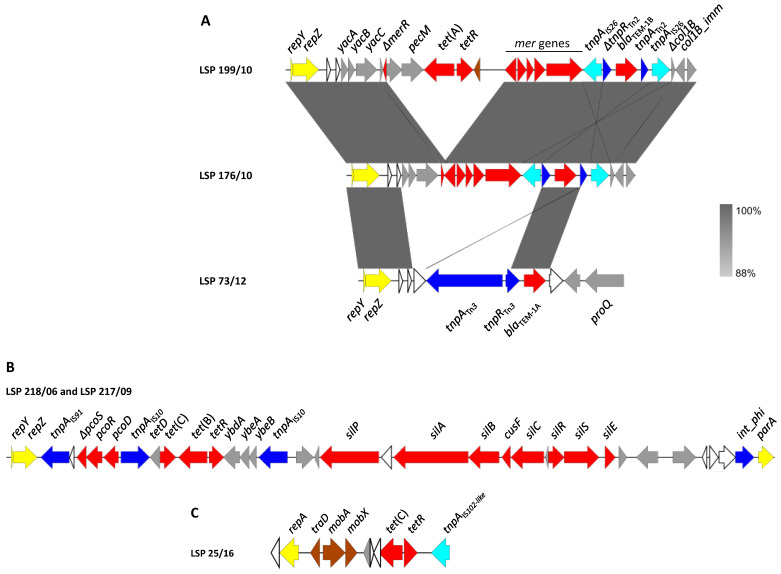
Genetic map of resistance genes carried by plasmids in *S*. Derby recovered from food and human clinical samples in Spain. Genes are represented by arrows pointing to the direction of transcription and color-coded according to function (see below). (**A**). Comparison of the resistance regions found in IncI1-I(α) plasmids of *bla*_TEM-1_-positive isolates. The alignments were created with Easyfig BLASTn. The gray shading between regions shows nucleotide sequence identities according to the scale shown at the right lower corner of the figure. (**B**). Resistance region of the *tet*(B)-positive IncI1-I(α) plasmids found in LSP 218/06 and LSP 217/09. (**C**). pSC101-like plasmid of LSP 25/16 used as a model. Color code: yellow, plasmid replication and maintenance; brown, conjugational transfer; blue, DNA mobility; red, resistance; grey, other functions; white, hypothetical proteins.

The genomic background of the resistance genes and their association with genetic elements potentially involved in DNA mobility were established by sequencing 22 isolates representing the diversity of resistance profiles. Resistance genes were either located on the chromosome or carried by different plasmids, usually as part of a class 1 integron or linked to insertion sequences or transposons (Table 2 and Figure 1 and Figure 2).

#### 3.3.1. Chromosomally Located Genes

In all isolates positive for *aadA2*, *sul1* and *tet*(A), with the profiles R6 to R9, these genes were clustered on the chromosome, as part of a ca. 43 kb region, absent in the susceptible isolates as well as in isolates with other resistance profiles. This region, which was identical or nearly identical in all isolates, has features consistent with SGI1 (Figure 1A, using LSP 138/08 as a model).

Like SGI1, the genomic island of the sequenced isolates was located between the *trmE* and *yidY* genes, carried the resistance region towards the *yidY* end and had a closely related backbone structure. However, the resistance region is remarkably different. Thus, instead of the In104 complex class 1 integron of SGI1, it contained a typical class 1 integron with the *intI1* gene, the 1000 bp/*aadA2* variable region and the 3′-conserved segment, where the *qacEΔ1* (for resistance to disinfectants) and *sul1* genes are located. The integron also carried the insertion sequence IS*1326* and a deleted *tni* module, followed by the *merEDACPTR* locus involved in mercury resistance. All these elements are characteristically associated with the class 1 integrons mobilized by Tn*21*-like transposons [35]. Indeed, one of the inverted repeats (IR) of this transposon was present in the analyzed region, but the other IR and the *tnpA* and *tnpR* genes, coding for the transposase and resolvase of Tn*21*, were missing. Apart from the class 1 integron, the SGI1-like element of *S.* Derby contained the *tet*(A) gene carried by a Tn*1721* remnant, and genes for restriction and modification enzymes, all absent in SGI1. An SGI1-like element identical to the one found in the current study, including the flanking DNA, has only been reported in two other *S.* Derby strains, CVM N17S1441 (accession number OK209937; [25]) and 2014LSAL02547 (accession number CP029486), associated with the pork sector in USA and France, respectively.

The genetic background of *fosA7.3* was also established for the sequenced isolates. In all of them, the gene occupied the same chromosomal location, which coincides with that reported for the *fosA7* gene of other serovars [36,37]. As shown in Figure 1B, multiple genes whose products are involved in DNA metabolism, including two HNH endonucleases, a class 1 DNA methyltransferase and two helicases, were found in the vicinity of *fosA7.3*. Interestingly, an insertion sequence, IS*102*-like, pertaining to the IS*903* group within the IS*5* family, was detected immediately downstream of the *fosA7.3* gene of one isolate (LSP 25/12). Other insertion sequences were previously found either upstream (IS*1351*) or downstream (IS*Ec1*) of *fosA7* [38].

#### 3.3.2. Plasmid-Located Genes

Additional resistance genes, including *bla*_TEM-1_, *tet*(A), *tet*(B) and *tet*(C), were carried either by IncI1-I(α) or pSC101-like plasmids (Figure 2).

IncI1-I(α) plasmids were found in six isolates (Table 2), one of them susceptible to all tested antimicrobials (LSP 101/16; profile R0), and the remaining five resistant to one or more antibiotics. A comparison of the resistance regions found in these plasmids and their flanking DNA is shown in Figure 2A. In the three ampicillin resistant isolates, LSP 176/10, LSP 199/10 and LSP 73/12 (profiles R4, R5 and R7, respectively), the *bla*_TEM-1_ gene was located on IncI1-I(α) plasmids assigned either to ST25 (LSP 176/10 and LSP 199/10) or to a new ST. The resistance modules of the three plasmids were placed downstream of *repZ*, the gene encoding the replication initiation protein of the IncI1-I(α) plasmids [39]. In contrast, the flanking DNA at the 3′-end was different. In the LSP 176/10 and LSP 199/10 plasmids, the resistance modules (ca. 6.6 and 11.5 kb, respectively) were located between *repZ* and a deleted gene encoding the pore-forming colicin Col1B, followed by the cognate immunity protein gene. The resistance module comprised truncated versions of the *mer* locus and of the Tn*2* transposon that carries *bla*_TEM-1B_. The latter was flanked by oppositely oriented copies of IS*26*, so the resulting pseudo-transposon could have been responsible for the deletions affecting both the *mer* locus and the colicin-encoding gene, located at each end. The resistance module of LSP 176/10, closely related to that of LSP 199/10, only differed by the absence of the *tet*(A) gene associated with a remnant of Tn*1721*, found in the latter isolate. On the other hand, the resistance module of the LSP 73/12 plasmid (ca. 5 kb) was embedded between *repZ* and the *proQ* gene, which encodes an RNA chaperon protein similar to FinOP repressors of conjugation of IncF plasmids [39]. This module just consisted of an intact Tn*3*·transposon where *bla*_TEM-1A_ was found. In agreement with straightforward transposition into the IncI1-I(α) backbone, the transposon was flanked by the expected five bp direct repeats. According to BLASTn comparisons, resistance regions identical to those found in the *bla*_TEM-1_-positive isolates reported herein were not previously detected in other IncI1-Iα plasmids, neither from *S. enterica* nor from any other bacteria.

The *tet*(B) gene of LSP 218/06 and LSP 217/09 (profile R1) was carried by yet another IncI1-I(α) plasmid with a new ST (allelic profile *ardA_3*, *pill_3*, *repI1_3*, *sogS_6*, *trbA_3*, according to pMLST), as part of a complex resistance region placed between *repZ* and a site-specific phage integrase gene (Figure 2B). Within this region (ca. 29.3 kp), which was nearly identical in the two isolates, the *tet*(B) gene was provided by a composite Tn*10* transposon flanked by two copies of IS*10*. Moreover, an insertion sequence belonging to the IS*91* family was found at the 5′-end of the resistance region, which also contained genes for copper and silver resistance. According to BLASTn comparisons, related regions were only carried by an IncI1 plasmid of *S.* Brandenburg (pSA20064858; accession number CP030003), or integrated into the chromosome of *S.* Bovismorbificans strain CVM 30176, probably by IS*10*-mediated transposition (accession number CP051349).

In the four isolates positive for *tet*(C) with the R2 (3) and R3 (1) profiles (Table 1 and Table 2), the gene was located on small plasmids of ca. 9.3 kb that differed by a maximum of four bp. These plasmids were nearly identical to pSC101, a low copy number plasmid originally detected in *S*. Typhimurium [40]; pSC101 is non-conjugative but can be mobilized by co-resident conjugative plasmids (accession number X01654; [41]). Closely related plasmids were found in two isolates of *S.* Anatum obtained from human feces and ground beef in USA (pSAN1-06-0624 and pSAN1-1175 of 9323 bp and 10,280 bp; accession numbers CP014660 and CP019898, respectively; [42]), but not in other *S. enterica* serovars. As shown in Figure 2C, using LSP 25/16 as a model, the *tet*(C) plasmids of the *S*. Derby isolates have a *repA* gene encoding a replication initiator protein of the *rep3* superfamily. They also contain the mobilization genes *mobA*, *mobX* and *traD*, with *mobA* and *traD* encoding the relaxase and the coupling protein (T4CP) of the conjugative type 4 secretion system. In addition, all carried an insertion sequence closely related with IS*102*, belonging to the IS*903* group within the IS*5* family. Upstream of IS*102*-like, the *tetR* and *tet*(C) genes, coding for the repressor and the efflux protein involved in tetracycline resistance, were located. It is of note that an IS*102*-like element was found adjacent to *fosA7.3* in the chromosome of one of the pSC101-like-positive isolates (LSP 25/12; Section 3.3.1). Therefore, the insertion sequence could have reached the new location through transposition from the plasmid. In agreement with this, the 9 bp direct repeats expected for the IS*903* group were found flanking the IS*102*-like in the chromosome. The 9 bps (GGTTGAGCG) were originally present at the insertion site, located in the intergenic region between *fosA7.3* and the endonuclease gene placed downstream (Figure 1B).

### 3.4. Phylogenetic Analysis

**Figure 3:**
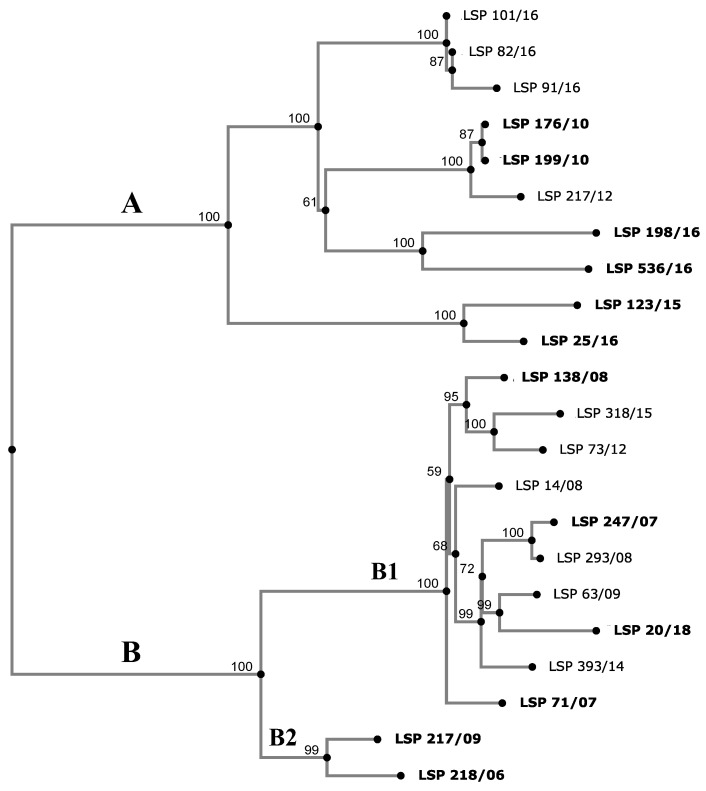
Phylogenetic tree showing the relationships between *S*. Derby isolates collected from food and human clinical samples in Spain. The SNP (single nucleotide polymorphism)-based tree was constructed with CSI Phylogeny 1.4 (https://cge.cbs.dtu.dk/services/CSIPhylogeny/, accessed on 10 May 2023), using the genome of *S*. Derby strain LSP 138/08 as the reference. Numbers at the nodes represent bootstrap values based on 1000 replicates. The observed clades (A and B) and subclades (B1 and B2) are indicated. The SNP similarity matrix is shown in Table S2. Human isolates are highlighted in bold.

To establish the relationships existing between the isolates, a phylogenetic tree was constructed based on SNPs detected by CSI Phylogeny in the genomes sequenced. The number of SNPs ranged from 2 (LSP 82/16 vs. LSP 101/16 and LSP 176/10 vs. LSP 199/10) up to 293 (LSP 198/16 vs. LSP 20/18). The isolates were distributed into 2 clades (A and B), both comprising food-borne and human clinical isolates and supported by 100% bootstrap (Figure 3).

Clade A grouped 10 isolates, which differed by 2 (LSP 82/16 and LSP 101/16) up to 195 (LSP 123/15 and LSP 198/16) SNPs. Three isolates, which were obtained from different samples consisting of mixed pork and beef minced meat, over a short period of time (LSP 82/16, LSP 91/16 and LSP 101/16), varied by a maximum of fourteen SNPs and were susceptible to all antibiotics tested (R0). Accordingly, they could belong to the same strain. Apart from R0, isolates with four other resistance profiles (R2, R3, R4 and R5) were shown to belong to clade A. Clade B contained the remaining 12 isolates, separated by 10 (LSP 293/08 and LSP 247/07) up to 141 (LSP 218/06 and LSP 20/18) SNPs. All except two isolates (LSP 218/06 and LSP 217/09) were MDR, showing the major resistance profile (R6) or variants of this which likely have arisen through acquisition of additional resistances (R7, R8 and R9). They were all closely related, differing by 10 to 78 SNPs (subcluster B1). LSP 218/06 and LSP 217/09 (subcluster B2), which only displayed *tet*(B)-mediated tetracycline resistance were closely related (R1 profile). They differed by only 40 SNPs and were separated from subclade B1 by a maximum of 135 and 141 SNPs, respectively.

## 4. Discussion

From 2006 to 2018, a total of 4310 isolates of *S. enterica* were recovered in Asturias; 3798 were identified at hospitals within the region as causes of human infections, while another 512 were collected from food samples. Most of them pertained to *S.* Enteritidis, *S.* Typhimurium and the monophasic 1,4,[5],12:i:-, a variant of the latter serovar, which are clearly predominant in the EU [3]. Taken together, they represented 79.5% and 57% of clinical and food isolates detected in Asturias, respectively. In contrast, only 20 (0.5%) clinical isolates and 16 (3.1%) food isolates belonged to *S.* Derby, and these were selected for the present study. The percentage of clinical *S.* Derby isolates in our region was somewhat lower than that reported for the EU, where this serotype accounted for 0.9% of the confirmed cases of human salmonellosis in 2018 [43]. Despite its relatively low frequency, *S.* Derby has consistently ranked as one of the most prevalent serovars of *S. enterica* over the last several years [3,43].

Interestingly, all sequenced *S*. Derby isolates detected in Asturias, including those causing human infections, belonged to ST40 which, together with ST39, forms one of the main lineages of this serovar. ST40 was also responsible for most human clinical cases of salmonellosis in France (71%), where two other pork-related STs (ST39 and ST682; 14.7% and 8.4%, respectively) and the poultry-associated ST71 (2%) were also involved [22]. The isolates sequenced herein were closely related, differing by a maximum of 293 SNPs. However, they were distributed into two distinct clades, A and B, both grouping clinical and food-borne isolates, consistent with their spread through the food chain. In addition, both contained resistant isolates, while all susceptible isolates belonged to clade A and all MDR isolates were grouped in clade B. Moreover, all SGI1-like positive isolates, with or without additional resistance genes, formed a coherent subclade within clade B. Two clades were also identified among pork and human ST40 isolates in France [22].

All but one of the food-borne isolates detected in Asturias derived from pig carcasses or pork meat-containing samples. Still, more than half of these samples (10 out of 16; 62.5%) consisted of mixed pork and beef minced meat, so the contribution of cattle to transmission of *S*. Derby into humans cannot be ruled out. In previous studies, ST40 isolates were predominantly associated with human clinical samples, pig-related samples including pig carcasses at slaughter, pork-derived products, the environment at slaughterhouses and, in some cases, isolates from the poultry industry, including turkey [13,14,18,19,20,23]. Nevertheless, information on the presence of *S.* Derby in cattle is rather limited. This has been attributed to the low prevalence of *S. enterica* in cattle and cattle meat in the EU, when compared with poultry, pigs and derived products [3,18]. However, 18.7% of the *S*. Derby genomes analyzed by González-Santamarina et al. in Germany were cattle isolates [18]. Although most of them were ST39, ST40 was also detected. One of the food isolates in the present study derived from wild boar. As far as we know only a single *S*. Derby from this source has been previously sequenced, and it was assigned to ST40 like the one reported herein [18]).

In the present study, 30.6% of the analyzed isolates (11/36) were susceptible to all antimicrobials tested. However, the *fosA7.3* gene of chromosomal location was detected in all that were sequenced. In gram-negative bacteria, the *fosA* gene, located either on the chromosome or carried by plasmids, is the main mechanism of resistance to fosfomycin, a broad-spectrum bactericidal antibiotic used to treat urinary tract infections, particularly those caused by ESBL (extended-spectrum β-lactamase) producers [44,45]. Up to now, three *fosA* alleles, *fosA3*, *fosA4* and *fosA7*, have been found in *S. enterica* [44]. The *fosA7* variant was first reported on the chromosome of *S*. Heidelberg recovered from broiler chickens in Canada [36], and then in several other serovars and STs, including S. Derby ST39, also on the chromosome [18,22,36,37,38]. In contrast to other *fosA* alleles, *fosA7* does not confer or only confers low level resistance to fosfomycin (MIC of 0.5–32 mg/L) [36,37,38]. Nevertheless, high level resistance (MIC ≥ 512 mg/L) could be achieved after transferring the gene, cloned onto high- or lower-copy number plasmid vectors, into different host bacteria, including several susceptible serovars of *S. enterica* [36,37]. Although not experimentally tested, this could also apply to *fosA7.3*, considering that the FosA7 and FosA7.3 proteins diverge by only five amino acids, with three of them being conservative substitutions. Thus, the presence of *fosA7* alleles, including *fosA7.3*, is of concern, because transferring of the gene from the chromosome into a coresident plasmid may led not only to high fosfomycin resistance but also to further spread of the gene.

It is well established that insertion sequences play a key role in the mobilization of resistance genes, and that they can enhance its expression [6]. The presence of IS*102*-like in the vicinity of the chromosomal *fosA7.3* gene of LSP 25/16 might propitiate the transfer of the gene from the chromosome into a plasmid. As indicated above, LSP 25/16 carried a pSC101-*tet*(C)-like plasmid where an IS*102*-like insertion sequence was already located, and which could have been the donor of the chromosomal IS. Once in the vicinity of *fosA7.3*, a further involvement of IS*102* in the mobilization of the resistance gene cannot be disregarded. In fact, evolution of a pSC101-like plasmid through IS*102*-like-mediated acquisition of additional resistance genes has previously been reported [46]. The resulting plasmid, pMC2, detected in agricultural soil after application of swine manure, contains the pSC101-*tet*(C) core, but acquired a IS*102*-based composite transposon, where macrolide, mercury and chromium resistance genes are located. Interestingly, LSP 25/16 was the only clinical isolate recovered from urine samples in our region. The patient, an 82-year-old female, was likely to have been treated with fosfomycin. Such treatment would exert a selective pressure for enhanced expression of the *fosA7.3* gene.

Besides *fosA7.3*, all isolates sequenced carried a cryptic *aac(6′)-1aa* gene. This gene is widespread in *S. enterica*, being present in the chromosome of multiple serovars. However, it does not confer resistance to kanamycin, tobramycin and amikacin, which is the phenotype expected for AAC(6′)-Iaa [47]. In vitro evolution experiments, aimed to assess the evolutionary potential of the silent *aac(6′)-1aa* of *S.* Typhimurium LT2 concluded, with a 99.99% confidence, that no single amino acid substitution or combination of two independent amino acid substitutions in *aac(6′)-Iaa* is capable of increasing resistance to any of the antibiotics used. In light of its inability to evolve either an increase in activity or an extended substrate specificity, it was concluded that *aac(6′)-Iaa*, even if mobilized to a plasmid, is unlikely to become a problem of clinical significance. However, a closely related *aac(6′)-1aa* gene, *aac(6′)-1γ*, found in *S*. Enteritidis (only differing by two amino acids), was shown to confer resistance to tobramycin in a mutant having a large chromosomal deletion which fused the cryptic gene to a strong promoter [48]. Moreover, the silent gene exhibited the resistance profile typical of an Aac(6‘)-I enzyme when the *aac(6′)-1γ* gene cloned onto a high copy number vector was expressed in *E. coli* [48]. Similarly, enhanced expression of the silent by nevertheless functional *aac(6′)-Iaa* gene could still led to aminoglycoside resistance in *S. enterica*.

The frequency of phenotypic resistance in *S*. Derby isolates from Asturias was 69.4%, being higher in human (85%; 17/20) than in food (50%; 8/16) isolates. Interestingly, all detected resistances were against traditional antibiotics, whereas resistance to those regarded as critically important in human medicine, such as third and fourth generation cephalosporins, carbapenems or colistin [49], was not observed.

As already indicated, six isolates proved to be resistant to nalidixic acid. Four of them carried the GAC to AAC mutation in *gyrA*, yielding the Asp87/Asn substitution in the protein. However, for two other isolates, specifically the only one recovered from wild boar (LSP 318/13) and another one of human origin (LSP 247/07), the genetic bases of nalidixic acid resistance were not identified. Plasmid-mediated quinolone resistance genes were not detected, but the possible involvement of efflux pumps was not investigated. The AAC into AGC substitution in *parC* (Thr57 to Ser in the protein) could not be the cause of nalidixic acid resistance, as it was present in both susceptible and resistant isolates. Therefore, it can be regarded as a polymorphism, as previously proposed for several other *S. enterica* serovars [50,51,52,53,54].

Consistent with previous findings, the highest rate of resistance was to streptomycin, sulfonamides and tetracycline, encoded by *aadA2*, *sul1* and *tet*(A) located on a chromosomal SGI1-like element, specific to ST40 [11,22,23]. This resistance profile was first identified in 2005 in the pork sector of Spain [16]. In Asturias, isolates with this profile were already detected in 2007 as causes of human infections (LSP 71/07 and LSP 107/07) and they were still circulating in 2018 (LSP 20/18). Similar isolates are widespread in humans and pork in several European countries, including France and Germany, as well as in the USA [14,18,25,55]. Together with antibiotic resistance genes, all SGI1-like positive isolates contained the entire *mer* locus of Tn*21* (*merRTPCADE*), adjacent to the class 1 integron with the 1000 bp/*aadA2* variable region. This integron is similar to that carried by SGI1-C, a SGI1 variant reported in *S*. Derby isolates collected from human clinical samples in the Netherlands [11]. However, neither the entire nucleotide sequence of SGI1-C nor that of its integron are available. Up to now, SGI1 variants nearly identical or closely related to those reported herein were only found in the chromosome of three other *S*. Derby strains. They contained either the same integron with *aadA2* in the variable region (strains CVM N17S1441 and 2014LSAL02547; accession numbers OK209937 and CP029486, respectively), or a different one with *dfrA12*-*aadA2* (strain CVM N18S0789; accession number OK209939; [25]), together with the *mer* locus. The temporal persistence and broad geographical spread of *S*. Derby ST40 isolates positive for SGI1-like elements has been attributed to the selective advantages conferred by the island, due to the encoded resistances to traditional antibiotics that are commonly used in the veterinary field, and also to mercury and disinfectants which can act as co-selecting agents [56,57,58].

Together with genomic islands, like SGI1, plasmid acquisition is another key feature in the evolution of *S. enterica* and its resistance properties. In the present study, a high diversity of IncI1-I(α) plasmids, assigned to distinct STs (ST25, a new ST nearest to ST278, and an entirely new ST) was identified as a source of resistance genes. IncI1 plasmids are widely distributed in *S. enterica* and other clinically relevant Enterobacterales and have a great capability to acquire and disseminate resistance genes. Indeed, they have been associated with many resistance genes, including the β-lactamases genes *bla*_TEM_, *bla*_CTX-M_, *bla*_SHV_ and *bla*_CMY_, and also genes for resistance to aminoglycosides, tetracycline and quinolones [59,60]. Specifically, *bla*_TEM-1_ and *tet*(B) were the resistance genes carried by IncI1-I(α) plasmids analyzed in the current study. Despite their ST diversity, all have the resistance regions inserted at the same position, downstream of *repZ*. This further supports the existence of a hot spot for the acquisition of accessory DNA by IncI1 plasmids, as previously reported [59]. In the present study, several transposable elements were apparently involved in the building of the resistance regions. These include Tn*2*, Tn*3*, Tn*10*, IS*26* and IS*91*. Interestingly, a IncI1-I(α) plasmid with yet another ST (ST134) was present in a susceptible isolate (LSP 101/16), which could be a precursor of new resistance plasmids.

Apart from the relatively large IncI1-I(α) plasmids containing *tet*(B), small pSC101-like plasmids, positive for IS*102*, also contributed to tetracycline resistance of our *S*. Derby isolates by supplying *tet*(C). pSC101 (9239 bp), first identified in *S.* Typhimurium [40,41], was also detected among tetracycline resistant bacteria found in the gut of organically reared adult pigs farmed in Scotland (UK), using a metagenomic approach [61]. As indicated before, a larger pSC101-*tet*(C) derivative carrying additional resistance genes was isolated from swine manure [46]. This highlights the capability of IS*102* to shape new and more complex resistance regions by mobilizing resistance genes.

## 5. Conclusions

Overall, the present study emphasizes the relevance of *S*. Derby ST40 as a food-borne zoonotic pathogen in the region studied. Contaminated pork is probably the main vehicle of transmission, but wild boar has also been implicated, and the possible involvement of cattle cannot be disregarded. Resistance to traditional antibiotics and heavy metals was shown to be conferred by a genomic island and two plasmid types (IncI1-Iα and pSC101-like), with a class 1 integron, multiple insertion sequences and several intact or truncated transposons shaping diverse resistance regions. The in-depth analysis of the genomic diversity of *S*. Derby in Asturias, and of the nature and origin of its resistance will be useful for further epidemiological surveillance and control.

## Figures and Tables

**Table 1:** Phenotypic resistance properties of *Salmonella enterica* serovar Derby isolates from different sources and the presence of genes as detected by PCR.

R-Profile (N) ^a^	R-Phenotype/R-Genes ^a,b^	Origin (N) ^a,c^	Isolates (LSP) ^d^
R0 (11)	Susceptible/-	PB (8), HF (3)	48/11, 496/14; 32/15, 33/15, **82/16**, 83/16, **91/16**, **101/16** 102/16, 103/16, **536/16**
R1 (2)	TET/*tet*(B)	HF (2)	**218/06**, **217/09**
R2 (3)	TET/*tet(*C)	HF (2), HU (1)	**123/15**, **25/16**, **198/16**
R3 (1)	TET NAL/*tet*(C) nd	PS (1)	**217/12**
R4 (1)	AMP NAL/*bla*_TEM-1B_ nd	HF (1)	**176/10**
R5 (1)	AMP TET NAL/*bla*_TEM-1B_ *tet*(A) nd	HF (1)	**199/10**
R6 (13)	STR SUL TET/*aadA2 sul1 tet*(A)	PC (2), PB (2), HF (9)	**71/07**, 107/07, 70/08, **138/08**, **293/08**, **63/09**, 20/10, 231/13, 270/13, 110/14, **393/14**, 396/14, **20/18**
R7 (1)	AMP STR SUL TET/*bla*_TEM-1A_ *aadA2 sul1 tet*(A)	PC (1)	**73/12**
R8 (1)	STR SUL TET NAL/*aadA2 sul1 tet*(A) nd	PS (1)	**14/08**
R9 (2)	STR SUL TET NAL/*aadA2 sul1 tet*(A) nd	WB (1), HF (1)	**247/07**, **318/15**

^a^ R, resistance; N, number of isolates. ^b^ AMP, ampicillin; STR, streptomycin; SUL, sulfonamides; TET, tetracycline; NAL, nalidixic acid; nd, not experimentally determined. A *fosA7*-like gene was detected in all isolates. ^c^ PB, pork and beef minced meat; PS, fresh pork sausage; PC, pig carcass; WB, wild boar fresh ground meat-derived product; HF, human feces; HU, human urine. ^d^ LSP, “Laboratorio de Salud Pública”, Asturias, Spain; sequenced isolates are highlighted in bold.

**Table 2:** Presence of resistance genes and genetic elements (integrons, transposons and plasmids) in *Salmonella enterica* serovar Derby isolates of different origins sequenced in the present study.

Isolate ^a^	Origin ^b^	R-Profile: Phenotype/R-Genes ^c^/In ^d^/Tn ^e^	Plasmid(s) ^f^
LSP 82/16	PB	R0: susceptible	-
LSP 91/16	PB	R0: susceptible	-
LSP 101/16	PB	R0: susceptible	IncI1-I(α)/ST134
LSP 536/16	HF	R0: susceptible	Col (pHAD28)
LSP 218/06	HF	R1: TET/*tet*(B)/Tn*10*	**IncI1-I(α)/uknST**
LSP 217/09	HF	R1: TET/*tet*(B)/Tn*10*	**IncI1-I(α)/uknST**
LSP 123/15	HF	R2: TET/*tet(*C)	**pSC101-like**
LSP 25/16	HU	R2: TET/*tet(*C)	**pSC101-like**
LSP 198/16	HF	R2: TET/*tet(*C)	**pSC101-like**
LSP 217/12	PS	R3: TET NAL/*tet*(C), *gyrA* (Asp87 to Asn)	**pSC101-like**, p0111, ColE10
LSP 176/10	HF	R4: AMP NAL/*bla*_TEM-1B,_ g*yrA* (Asp87 to Asn)/ΔTn*2*	**IncI1-I(α)/ST25**, p0111
LSP 199/10	HF	R5: AMP TET NAL/*bla*_TEM-1B_ *tet*(A) *gyrA* (Asp87 to Asn) /ΔTn*2/*ΔTn*1721*	**IncI1-1(α)/ST25**, p0111
LSP 71/07	HF	R6: STR SUL TET/*aadA2 sul1 tet*(A)/ + /ΔTn*1721*	IncQ1
LSP 138/08	HF	R6: STR SUL TET/*aadA2 sul1 tet*(A)/ + /ΔTn*1721*	-
LSP 293/08	PC	R6: STR SUL TET/*aadA2 sul1 tet*(A)/ + /ΔTn*1721*	-
LSP 63/09	PC	R6: STR SUL TET/*aadA2 sul1 tet*(A)/ + /ΔTn*1721*	IncY
LSP 393/14	PB	R6: STR SUL TET/*aadA2 sul1 tet*(A)/ + /ΔTn*1721*	ColE
LSP 20/18	HF	R6: STR SUL TET/*aadA2 sul1 tet*(A)/ + /ΔTn*1721*	-
LSP 73/12	PC	R7: AMP STR SUL TET/*bla*_TEM-1A_ *aadA2 sul1 tet*(A) / + */*ΔTn*1721/*Tn*3*	**IncI1-I(α)/nST278**
LSP 14/08	PS	R8: STR SUL TET NAL/*aadA2 sul1 tet*(A) *gyrA* (Asp87 to Asn) / + /ΔTn*1721*	IncP, ColE
LSP 247/07	HF	R9: STR SUL TET NAL/*aadA2 sul1 tet*(A) ni/ + /ΔTn*1721*	-
LSP 318/15	WB	R9: STR SUL TET NAL/*aadA2 sul1 tet*(A) ni/ + /ΔTn*1721*	-

^a^ LSP, “Laboratorio de Salud Pública”, Asturias, Spain. ^b^ PB, pork and beef minced meat; PS, fresh pork sausage; PC, pig carcass; WB, fresh wild boar ground meat-derived product; HF, human feces; HU, human urine. ^c^ R, resistance; AMP, ampicillin; STR, streptomycin; SUL, sulfonamides; TET, tetracycline; NAL, nalidixic acid; Δ, deleted; ni, not identified. The *aac(6′)-1aa* gene, the *fosA7.3* genes, and the Thr57 to Ser substitution in ParC were found in all sequenced isolates, although they were not associated with resistance. ^d^ +, presence of a class 1 integron (In) with *aadA2* in the variable region and *sul1* in the 3′-conserved segment; ^e^ Tn, transposon; ^f^ Inc, incompatibility group; n, new; ukn, unknown. Resistance plasmids are shown in bold.

## Data Availability

The genome sequences generated in the present study were deposited at GenBank database under BioProject PRJNA911783, with the following accession numbers JAPWTI000000000, JAPWTA000000000, JAPWTF000000000, JAPWSX000000000, JAPWTD000000000, JAPWSZ000000000, JASTWD000000000, JAPWTG000000000, JAPWTE000000000, JASNGD000000000, JAWGG000000000, JAPWGB000000000, JASJEL000000000, JAPWTC000000000, JAPWTH000000000, JAWSY000000000, JAPWGF000000000, JAPWGE000000000, JAPWTB000000000, JAPWGC000000000, JASIRX000000000 and JASIRW000000000 for LSP 218/06, LSP 71/07, LSP 247/07, LSP 14/08, LSP 138/08, LSP 63/09, LSP 217/09, LSP 293/09, LSP 176/10, LSP 199/10, LSP 73/12, LSP 217/12, LSP 393/13, LSP 123/15, LSP 318/15, LSP 25/16, LSP 82/16, LSP 91/16, LSP 101/16, LSP 198/16, LSP 356/16 and LSP 20/18, respectively.

## Supplementary Materials

The following supporting information can be downloaded at: https://www.mdpi.com/article/10.3390/antibiotics12071204/s1, Table S1: Primers used for detection of resistance genes, amplification conditions and size of expected amplicons [28,29,30,62,63,64,65,66,67]. Table S2: Accession numbers of *Salmonella enterica* serovar Derby isolates obtained from food and clinical samples in Spain, and parameters related to the quality of the assemblies. Table S3: Pairwise distance matrix calculated from SNP in the genomes of *Salmonella enterica* serovar Derby isolates obtained from food and clinical samples in Spain. Figure S1: Agarose gels showing examples of PCR fragments amplified with primer pairs specific for the following resistance genes: *fosA7*, *aadA2*, *bla*_TEM-1_-like, *sul1*, *tet*(A), *tet*(B) and *tet*(C), with the size of the expected amplicons shown in parenthesis. Figure S2: Plasmid profiles of *Salmonella enterica* serovar Derby isolates harbouring resistance genes of plasmid location [68,69,70].

## References

[1] Majowicz S.E., Musto J., Scallan E., Angulo F.J., Kirk M., O’Brien S.J., Jones T.F., Fazil A., Hoekstra R.M., International Collaboration on Enteric Disease ‘Burden of Illness’ Studies (2010). The global burden of nontyphoidal *Salmonella* gastroenteritis. Clin. Infect. Dis..

[2] WHO (2018). (World Health Organization): Salmonella (Non-Typhoidal)—Fact Sheet. http://www.who.int/mediacentre/factsheets/fs139/en/.

[3] European Food Safety Authority, European Centre for Disease Prevention and Control (2022). The European Union One Health 2021 Zoonoses Report. EFSA J..

[4] WHO (2015). (World Health Organization): Global Action Plan on Antimicrobial Resistance. World Health Organization. https://www.who.int/publications/i/item/9789241509763.

[5] Grimont P.A.D., Weill F.X. (2007). Antigenic formulae of the *Salmonella* serovars. World Health Organization Collaborating Center for Reference and Research on Salmonella.

[6] Partridge S.R., Kwong S.M., Firth N., Jensen S.O. (2018). Mobile genetic elements associated with antimicrobial resistance. Clin. Microbiol. Rev..

[7] Threlfall E.J. (2000). Epidemic *Salmonella typhimurium* DT 104—A truly international multiresistant clone. J. Antimicrob. Chemother..

[8] Boyd D., Peters G.A., Cloeckaert A., Boumedine K.S., Chaslus-Dancla E., Imberechts H., Mulvey M.R. (2001). Complete nucleotide sequence of a 43-kilobase genomic island associated with the multidrug resistance region of *Salmonella enterica* serovar Typhimurium DT104 and its identification in phage type DT120 and serovar Agona. J. Bacteriol..

[9] Hall R.M. (2010). *Salmonella* genomic islands and antibiotic resistance in *Salmonella enterica*. Future Microbiol..

[10] Mulvey M.R., Boyd D.A., Olson A.B., Doublet B., Cloeckaert A. (2006). The genetics of *Salmonella* genomic island 1. Microbes Infect..

[11] Beutlich J., Jahn S., Malorny B., Hauser E., Huhn S., Schroeter A., Rodicio M.R., Appel B., Threlfall J., Mevius D. (2011). Antimicrobial resistance and virulence determinants in European *Salmonella* genomic island 1-positive *Salmonella enterica* isolates from different origins. Appl. Environ. Microbiol..

[12] Cummins M.L., Hamidian M., Djordjevic S.P. (2020). *Salmonella* genomic island 1 is broadly disseminated within Gammaproteobacteriaceae. Microorganisms.

[13] Hauser E., Hebner F., Tietze E., Helmuth R., Junker E., Prager R., Schroeter A., Rabsch W., Fruth A., Malorny B. (2011). Diversity of *Salmonella enterica* serovar Derby isolated from pig, pork and humans in Germany. Int. J. Food Microbiol..

[14] Kerouanton A., Rose V., Weill F.X., Granier S.A., Denis M. (2013). Genetic diversity and antimicrobial resistance profiles of *Salmonella enterica* serotype Derby isolated from pigs, pork, and humans in France. Foodborne Pathog. Dis..

[15] Michael G.B., Cardoso M., Rabsch W., Schwarz S. (2006). Phenotypic and genotypic differentiation of porcine *Salmonella enterica* subsp. *enterica* serovar Derby isolates. Vet. Microbiol..

[16] Valdezate S., Vidal A., Herrera-León S., Pozo J., Rubio P., Usera M.A., Carvajal A., Echeita M.A. (2005). *Salmonella* Derby clonal spread from pork. Emerg. Infect. Dis..

[17] Simon S., Trost E., Bender J., Fuchs S., Malorny B., Rabsch W., Prager R., Tietze E., Flieger A. (2018). Evaluation of WGS based approaches for investigating a food-borne outbreak caused by *Salmonella enterica* serovar Derby in Germany. Food Microbiol..

[18] González-Santamarina B., García-Soto S., Hotzel H., Meemken D., Fries R., Tomaso H. (2021). *Salmonella* Derby: A comparative genomic analysis of strains from Germany. Front. Microbiol..

[19] Hayward M.R., Jansen V., Woodward M.J. (2013). Comparative genomics of *Salmonella enterica* serovars Derby and Mbandaka, two prevalent serovars associated with different livestock species in the UK. BMC Genom..

[20] Hayward M.R., Petrovska L., Jansen V.A., Woodward M.J. (2016). Population structure and associated phenotypes of *Salmonella enterica* serovars Derby and Mbandaka overlap with host range. BMC Microbiol..

[21] Sevellec Y., Felten A., Radomski N., Granier S.A., Le Hello S., Petrovska L., Mistou M.Y., Cadel-Six S. (2019). Genetic diversity of *Salmonella* Derby from the poultry sector in Europe. Pathogens..

[22] Sevellec Y., Granier S.A., Le Hello S., Weill F.X., Guillier L., Mistou M.Y., Cadel-Six S. (2020). Source attribution study of sporadic *Salmonella* Derby cases in France. Front. Microbiol..

[23] Sevellec Y., Vignaud M.L., Granier S.A., Lailler R., Feurer C., Le Hello S., Mistou M.Y., Cadel-Six S. (2018). Polyphyletic nature of *Salmonella enterica* serotype Derby and lineage-specific host-association revealed by genome-wide analysis. Front. Microbiol..

[24] Hayward M.R., AbuOun M., La Ragione R.M., Tchorzewska M.A., Cooley W.A., Everest D.J., Petrovska L., Jansen V.A., Woodward M.J. (2014). SPI-23 of *S*. Derby: Role in adherence and invasion of porcine tissues. PLoS ONE.

[25] Li C., Tyson G.H., Hsu C.H., Harrison L., Strain E., Tran T.T., Tillman G.E., Dessai U., McDermott P.F., Zhao S. (2021). Long-read sequencing reveals evolution and acquisition of antimicrobial resistance and virulence genes in *Salmonella enterica*. Front. Microbiol..

[26] CLSI (2019). Performance Standards for Antimicrobial Susceptibility Testing.

[27] Magiorakos A.P., Srinivasan A., Carey R.B., Carmeli Y., Falagas M.E., Giske C.G., Harbarth S., Hindler J.F., Kahlmeter G., Olsson-Liljequist B. (2012). Multidrug-resistant, extensively drug-resistant and pandrug-resistant bacteria: An international expert proposal for interim standard definitions for acquired resistance. Clin. Microbiol. Infect..

[28] Guerra B., Junker E., Miko A., Helmuth R., Mendoza M.C. (2004). Characterization and localization of drug resistance determinants in multidrug-resistant, integron-carrying *Salmonella enterica* serotype Typhimurium strains. Microb. Drug Resist..

[29] Ng L.K., Mulvey M.R., Martin I., Peters G.A., Johnson W. (1999). Genetic characterization of antimicrobial resistance in Canadian isolates of *Salmonella* serovar Typhimurium DT104. Antimicrob. Agents Chemother..

[30] Perreten V., Boerlin P. (2003). A new sulfonamide resistance gene (*sul3*) in *Escherichia coli* is widespread in the pig population of Switzerland. Antimicrob. Agents Chemother..

[31] Bankevich A., Nurk S., Antipov D., Gurevich A.A., Dvorkin M., Kulikov A.S., Lesin V.M., Nikolenko S.I., Pham S., Prjibelski A.D. (2012). SPAdes: A new genome assembly algorithm and its applications to single-cell sequencing. J. Comput. Biol..

[32] Tatusova T., DiCuccio M., Badretdin A., Chetvernin V., Nawrocki E.P., Zaslavsky L., Lomsadze A., Pruitt K.D., Borodovsky M., Ostell J. (2016). NCBI prokaryotic genome annotation pipeline. Nucleic Acids Res..

[33] Sullivan M.J., Petty N.K., Beatson S.A. (2011). Easyfig: A genome comparison visualizer. Bioinformatics.

[34] Felsenstein J. (1985). Confidence limits on phylogenies: An approach using the bootstrap. Evolution.

[35] Liebert C.A., Hall R.M., Summers A.O. (1999). Transposon Tn*21*, flagship of the floating genome. Microbiol. Mol. Biol. Rev..

[36] Rehman M.A., Yin X., Persaud-Lachhman M.G., Diarra M.S. (2017). First detection of a fosfomycin resistance gene, *fosA7*, in *Salmonella enterica* serovar Heidelberg isolated from broiler chickens. Antimicrob. Agents Chemother..

[37] Wang J., Wang Y., Wang Z.Y., Wu H., Mei C.Y., Shen P.C., Pan Z.M., Jiao X. (2021). Chromosomally located *fosA7* in *Salmonella* isolates from China. Front. Microbiol..

[38] Wang D., Fang L.X., Jiang Y.W., Wu D.S., Jiang Q., Sun R.Y., Wang M.G., Sun J., Liu Y.H., Liao X.P. (2022). Comparison of the prevalence and molecular characteristics of *fosA3* and *fosA7* among *Salmonella* isolates from food animals in China. J. Antimicrob. Chemother..

[39] Sampei G., Furuya N., Tachibana K., Saitou Y., Suzuki T., Mizobuchi K., Komano T. (2010). Complete genome sequence of the incompatibility group I1 plasmid R64. Plasmid..

[40] Cohen S.N., Chang A.C. (1977). Revised interpretation of the origin of the pSC101 plasmid. J. Bacteriol..

[41] Bernardi A., Bernardi F. (1984). Complete sequence of pSC101. Nucleic Acids Res..

[42] Nguyen S.V., Harhay D.M., Bono J.L., Smith T.P., Fields P.I., Dinsmore B.A., Santovenia M., Kelley C.M., Wang R., Bosilevac J.M. (2016). Complete and closed genome sequences of 10 *Salmonella enterica* subsp. *enterica* serovar Anatum isolates from human and bovine sources. Genome Announc..

[43] European Food Safety Authority, European Centre for Disease Prevention and Control (2021). The European Union One Health 2020 Zoonoses Report. EFSA J..

[44] Ito R., Mustapha M.M., Tomich A.D., Callaghan J.D., McElheny C.L., Mettus R.T., Shanks R.M.Q., Sluis-Cremer N., Doi Y. (2017). Widespread fosfomycin resistance in Gram-negative bacteria attributable to the chromosomal *fosA* gene. mBio..

[45] Sastry S., Doi Y. (2016). Fosfomycin: Resurgence of an old companion. J. Infect. Chemother..

[46] Rahube T.O., Yost C.K. (2012). Characterization of a mobile and multiple resistance plasmid isolated from swine manure and its detection in soil after manure application. J. Appl. Microbiol..

[47] Salipante S.J., Hall B.G. (2003). Determining the limits of the evolutionary potential of an antibiotic resistance gene. Mol. Biol. Evol..

[48] Magnet S., Courvalin P., Lambert T. (1999). Activation of the cryptic *aac(6′)-Iy* aminoglycoside resistance gene of *Salmonella* by a chromosomal deletion generating a transcriptional fusion. J. Bacteriol..

[49] WHO (2019). Critically Important Antimicrobials for Human Medicine. License: CC BY-NC-SA 30 IGO.

[50] Baucheron S., Chaslus-Dancla E., Cloeckaert A., Chiu C.H., Butaye P. (2005). High-level resistance to fluoroquinolones linked to mutations in *gyrA*, *parC*, and *parE* in *Salmonella enterica* serovar Schwarzengrund isolates from humans in Taiwan. Antimicrob. Agents Chemother..

[51] Kim K.Y., Park J.H., Kwak H.S., Woo G.J. (2011). Characterization of the quinolone resistance mechanism in foodborne *Salmonella* isolates with high nalidixic acid resistance. Int. J. Food Microbiol..

[52] Vázquez X., Fernández J., Hernáez S., Rodicio R., Rodicio M.R. (2022). Plasmid-mediated quinolone resistance (PMQR) in two clinical strains of *Salmonella enterica* serovar Corvallis. Microorganisms..

[53] Vázquez X., García P., García V., de Toro M., Ladero V., Heinisch J.J., Fernández J., Rodicio R., Rodicio M.R. (2021). Genomic analysis and phylogenetic position of the complex IncC plasmid found in the Spanish monophasic clone of *Salmonella enterica* serovar Typhimurium. Sci. Rep..

[54] Weill F.X., Bertrand S., Guesnier F., Baucheron S., Cloeckaert A., Grimont P.A. (2006). Ciprofloxacin-resistant *Salmonella* Kentucky in travelers. Emerg. Infect. Dis..

[55] Bonardi S. (2017). *Salmonella* in the pork production chain and its impact on human health in the European Union. Epidemiol. Infect..

[56] Baker-Austin C., Wright M.S., Stepanauskas R., McArthur J.V. (2006). Co-selection of antibiotic and metal resistance. Trends Microbiol..

[57] Deng W., Quan Y., Yang S., Guo L., Zhang X., Liu S., Chen S., Zhou K., He L., Li B. (2018). Antibiotic resistance in *Salmonella* from retail foods of animal origin and its association with disinfectant and heavy metal resistance. Microb. Drug Resist..

[58] Newell D.G., Koopmans M., Verhoef L., Duizer E., Aidara-Kane A., Sprong H., Opsteegh M., Langelaar M., Threfall J., Scheutz F. (2010). Food-borne diseases—The challenges of 20 years ago still persist while new ones continue to emerge. Int. J. Food Microbiol..

[59] Johnson T.J., Shepard S.M., Rivet B., Danzeisen J.L., Carattoli A. (2011). Comparative genomics and phylogeny of the IncI1 plasmids: A common plasmid type among porcine enterotoxigenic *Escherichia coli*. Plasmid.

[60] Rozwandowicz M., Brouwer M.S.M., Fischer J., Wagenaar J.A., Gonzalez-Zorn B., Guerra B., Mevius D.J., Hordijk J. (2018). Plasmids carrying antimicrobial resistance genes in Enterobacteriaceae. J. Antimicrob. Chemother..

[61] Kazimierczak K.A., Scott K.P., Kelly D., Aminov R.I. (2009). Tetracycline resistome of the organic pig gut. Appl. Environ. Microbiol..

[62] Sandvang D., Aarestrup F.M., Jensen L.B. (1997). Characterisation of integrons and antibiotic resistance genes in Danish multiresistant *Salmonella enterica* Typhimurium DT104. FEMS Microbiol. Lett..

[63] Walker R.A., Lindsay E., Woodward M.J., Ward L.R., Threlfall E.J. (2001). Variation in clonality and antibiotic-resistance genes among multirresistant *Salmonella enterica* serotype Typhimurium phage-type U302 (MR U302) from humans, animals and foods. Microb. Drug Resist..

[64] Madsen L., Aarestrup F.M., Olsen J.E. (2000). Characterisation of streptomycin resistance determinants in Danish isolates of *Salmonella* Typhimurium. Vet. Microbiol..

[65] Arlet G., Phillippon A. (1991). Construction by polymerase chain reaction and intragenic DNA probes for three main types of transferable β-lactamases (TEM, SHV, CARB). FEMS Microbiol. Lett..

[66] Chu C., Chiu C.H., Wu W.Y., Chu C.H., Liu T.P., Ou J.T. (2001). Large drug resistance virulence plasmids of clinical isolates of *Salmonella enterica* serovar Choleraesuis. Antimicrob. Agents Chemother..

[67] Ng L.K., Martin I., Alfa M., Mulvey M. (2001). Multiplex PCR for the detection of tetracycline resistant genes. Mol. Cell Probes..

[68] Kado C.I., Liu S.T. (1981). Rapid procedure for detection and isolation of large and small plasmids. J. Bacteriol..

[69] Threlfall E.J., Rowe B., Ferguson J.L., Ward L.R. (1986). Characterization of plasmids conferring resistance to gentamicin and apramycin in strains of *Salmonella typhimurium* phage type 204c isolated in Britain. J Hyg..

[70] Sánchez F., Jiménez G., Aguilar A., Baquero F., Rubio V. (1986). Plasmid pVA517C from *Escherichia coli* V517 is required for the expression of an antibiotic microcin. J. Antibiot..

