# Supplementary material for: Incidence and Genomic Background of Antibiotic Resistance in Food-Borne and Clinical Isolates of Salmonella enterica Serovar Derby from Spain
Source: Antibiotics (Basel). 2023 Jul 19;12(7):1204. doi: 10.3390/antibiotics12071204 (PMC10376468; doi:10.3390/antibiotics12071204)
Supplement: Supplementary file 1 [file antibiotics-12-01204-s001.zip › antibiotics-2495317-supplementary.pdf]

antibiotics

Supplementary material

Incidence and genomic background of antibiotic resistance in food-borne and clinical isolates of *Salmonella enterica* serovar Derby from Spain

Table S1. Primers used for detection of resistance genes, amplification conditions and size of expected amplicons.

Target	Sequence 5'-3'	Tm	Amplicon (bp)	Reference
aadA1-like	GTGGATGGCGGCCTGAAGCC/ ATTGCCCAGTCGGCAGCG	70	526	[62]
aadA2	TGTTGGTTACTGTGGCCGTA/ GATCTCGCCTTTCACAAAGC	56	623	[63]
strA	CTTGGTGATAACGGCAATTC/ CCAATCGCAGATAGAAGGC	60	669	[64]
strB	ATCGTCAAGGGATTGAAACC/ GGATCGTAGAACATATTGGC	60	509	[64]
*bla*TEM-1-like	TTGGGTGCACGAGTGGGT/ TAATTGTTGCCGGGAAGC	55	503	[65]
*bla*OXA-1-like	AGCAGCGCCAGTGCATCA/ ATTCGACCCCAAGTTTCC	60	708	[28]
*bla*PSE-1	CGCTTCCCGTTAACAAGTAC/ CTGGTTCATTTCAGATAGCG	65	419	[62]
fosA7	CAGTCGGGATGCTAAATCTC/ GATACTGGCGCTTACCTTAC	58	267	This study
sul1	CTTCGATGAGAGCCGGCGGC/ GCAAGGCGGAAACCCGCGCC	65	436	[62]

sul2	TCAACATAACCTCGGACAGT/ GATGAAGTCAGCTCCACCT	55	707	[66]
sul3	GAGCAAGATTTTTGGAATCG/ CTAACCTAGGGCTTTGGA	50	773	[30]
tet(A)	GCTACATCCTGCTTGCCTTC/ CATAGATCGCCGTGAAGAGG	55	210	[67]
tet(B)	TTGGTTAGGGGCAAGTTTTG/ GTAATGGGCCAATAACACCG	55	659	[67]
tet(C)	CTTGAGAGCCTTCAACCCAG/ ATGGTCGTCATCTACCTGCC	55	428	[67]
tet(G)	GCTCGGTGGTATCTCTGC/ AGCAACAGAATCGGGAAC	55	500	[28]
tet(M)	GTGGACAAAGGTACAACGAG/ CGGTAAAGTTCGTCACACAC	57	406	[29]

antibiotics

MDPI

Table S2. Accession numbers of *Salmonella enterica* serovar Derby isolates obtained from food and clinical samples in Spain, and parameters related to the quality of the assemblies.

Isolate[a]	Kmer	Contigs	N50	Longest contig (bp)	Total bp in contigs	Contigs > 1 kb	GenBank accession number
LSP 218/06	127	93	397161	685719	4979598	39	JAPWTI000000000
LSP 71/07	127	85	473222	1641246	4834536	29	JAPWTA000000000
LSP 247/07	127	83	473406	1480285	4835929	25	JAPWTF000000000
LSP 14/08	127	101	375436	897376	4843242	36	JAPWSX000000000
LSP 138/08	127	124	487459	1641081	4855858	35	JAPWTD000000000
LSP 63/09	127	103	414700	952109	4914147	34	JAPWSZ000000000
LSP 217/09	127	111	165660	707665	5076596	53	JASTWD000000000
LSP 293/09	127	77	473422	860081	4834348	24	JAPWTG000000000
LSP 176/10	127	88	317905	317905	4951122	31	JAPWTE000000000
LSP 199/10	127	85	409282	1200751	4973614	33	JASNGD000000000
LSP 73/12	127	233	76664	214620	4907238	147	JAPWGG000000000
LSP 217/12	127	236	60780	272135	4878792	164	JAPWGB000000000
LSP 393/13	127	78	473343	860016	4843491	28	JASJEL000000000
LSP 123/15	127	83	457230	457230	4825116	28	JAPWTC000000000
LSP 318/15	127	87	462555	1541386	4921753	30	JAPWTH000000000
LSP 25/16	127	81	326393	605518	4805373	31	JAPWSY000000000
LSP 82/16	127	233	64096	242078	4769093	157	JAPWGF000000000
LSP 91/16	127	272	59058	244242	4756890	185	JAPWGE000000000
LSP 101/16	127	111	537578	1477530	4904217	35	JAPWTB000000000
LSP 198/16	127	243	75300	241746	4768307	163	JAPWGC000000000
LSP 356/16	127	84	276140	599614	4832529	31	JASIRX000000000
LSP 20/18	127	71	473481	1641750	4840526	23	JASIRW000000000

[a], LSP, "Laboratorio de Salud Pública", Asturias, Spain.

antibiotics

MDPI

Table S3. Pairwise distance matrix calculated from SNP in the genomes of *Salmonella enterica* serovar Derby isolates obtained from food and clinical samples in Spain.

	LSP 101/16	LSP 123/15	LSP 138/08	LSP 14/08	LSP 176/10	LSP 198/16	LSP 199/10	LSP 20/18	LSP 217/09	LSP 217/12	LSP 218/06	LSP 247/07	LSP 25/16	LSP 293/08	LSP 318/15	LSP 393/14	LSP 536/16	LSP 63/09	LSP 71/07	LSP 73/12	LSP 82/16	LSP 91/16
**LSP 101/16**	0	161	237	236	93	120	93	263	214	102	220	251	150	247	253	245	115	246	238	248	2	12
**LSP 123/15**	161	0	264	263	170	195	170	290	241	179	247	278	55	274	280	272	192	273	265	275	163	173
**LSP 138/08**	237	264	0	35	246	267	246	62	109	253	115	50	253	46	42	44	266	45	37	37	239	249
**LSP 14/08**	236	263	35	0	243	266	243	57	108	250	114	45	252	41	51	39	265	40	36	46	238	248
**LSP 176/10**	93	170	246	243	0	129	2	272	223	21	229	260	159	256	262	254	124	255	247	257	95	105
**LSP 198/16**	120	195	267	266	129	0	129	293	244	138	250	281	184	277	283	275	103	276	268	278	122	130
**LSP 199/10**	93	170	246	243	2	129	0	272	223	21	229	260	159	256	262	254	124	255	247	257	95	105
**LSP 20/18**	263	290	62	57	272	293	272	0	135	279	141	58	279	54	78	52	292	43	63	73	265	275
**LSP 217/09**	214	241	109	108	223	244	223	135	0	230	40	123	230	119	125	117	243	118	110	120	216	226
**LSP 217/12**	102	179	253	250	21	138	21	279	230	0	236	267	168	263	269	261	133	262	254	264	104	114
**LSP 218/06**	220	247	115	114	229	250	229	141	40	236	0	129	236	125	131	123	249	124	116	126	222	232
**LSP 247/07**	251	278	50	45	260	281	260	58	123	267	129	0	267	10	66	40	280	41	51	61	253	263
**LSP 25/16**	150	55	253	252	159	184	159	279	230	168	236	267	0	263	269	261	181	262	254	264	152	162
**LSP 293/08**	247	274	46	41	256	277	256	54	119	263	125	10	263	0	62	36	276	37	47	57	249	259
**LSP 318/15**	253	280	42	51	262	283	262	78	125	269	131	66	269	62	0	60	282	61	53	37	255	265
**LSP 393/14**	245	272	44	39	254	275	254	52	117	261	123	40	261	36	60	0	274	35	45	55	247	257
**LSP 536/16**	115	192	266	265	124	103	124	292	243	133	249	280	181	276	282	274	0	275	267	277	117	125
**LSP 63/09**	246	273	45	40	255	276	255	43	118	262	124	41	262	37	61	35	275	0	46	56	248	258
**LSP 71/07**	238	265	37	36	247	268	247	63	110	254	116	51	254	47	53	45	267	46	0	48	240	250
**LSP 73/12**	248	275	37	46	257	278	257	73	120	264	126	61	264	57	37	55	277	56	48	0	250	260
**LSP 82/16**	2	163	239	238	95	122	95	265	216	104	222	253	152	249	255	247	117	248	240	250	0	14
**LSP 91/16**	12	173	249	248	105	130	105	275	226	114	232	263	162	259	265	257	125	258	250	260	14	0

min: 2 max: 293; SNP, Single Nucleotide Polymorphism; LSP, "Laboratorio de Salud Pública", Asturias, Spain.

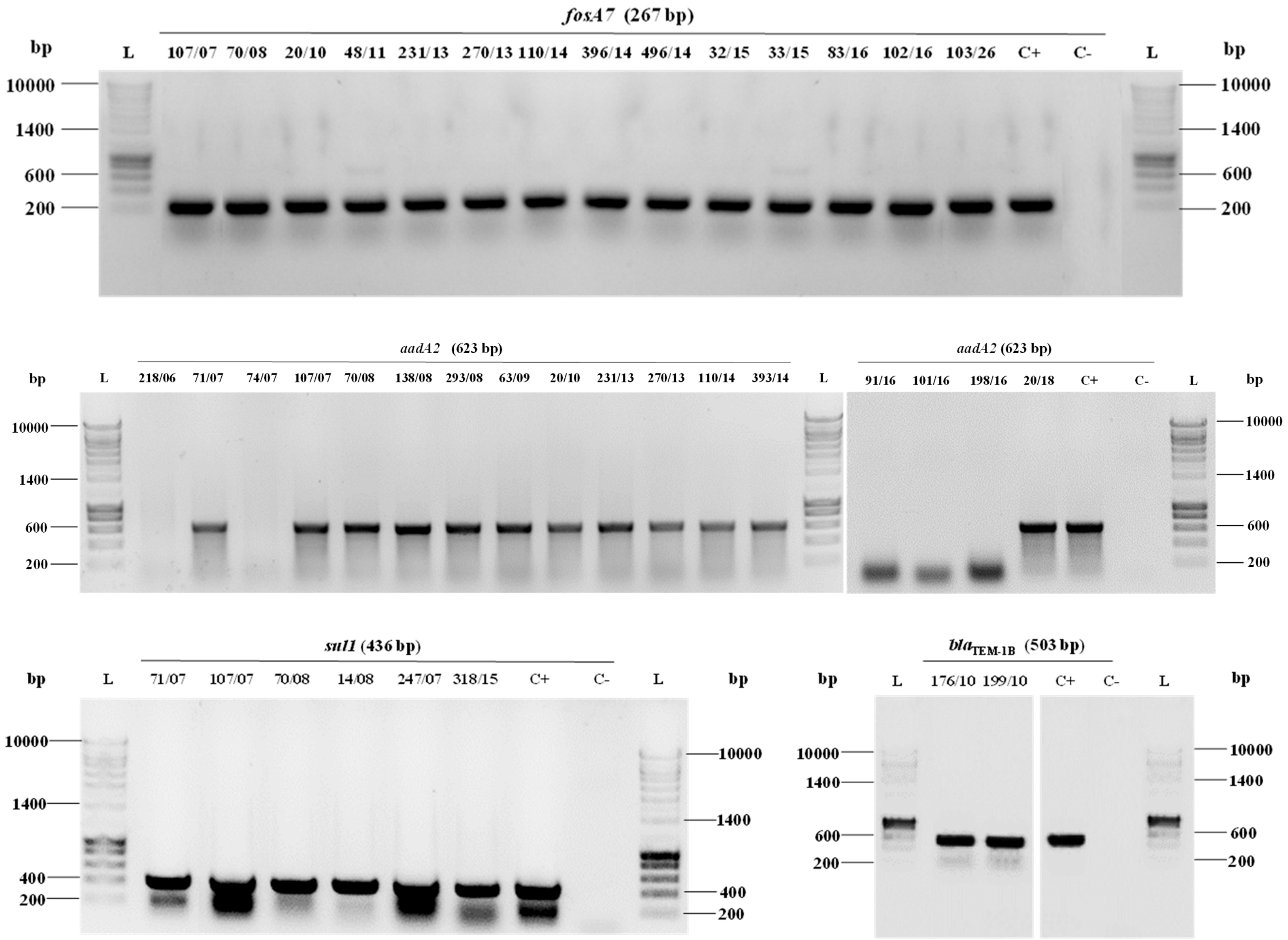

fosA7 (267 bp)
bp
L
107/07 70/08 20/10 48/11 231/13 270/13 110/14 396/14 496/14 32/15 33/15 83/16 102/16 103/26 C+ C-
10000
1400
600
200
aadA2 (623 bp)
218/06 71/07 74/07 107/07 70/08 138/08 293/08 63/09 20/10 231/13 270/13 110/14 393/14
91/16 101/16 198/16 20/18 C+ C-
sul1 (436 bp)
71/07 107/07 70/08 14/08 247/07 318/15 C+ C-
400
blaTEM-1B (503 bp)
176/10 199/10 C+ C-

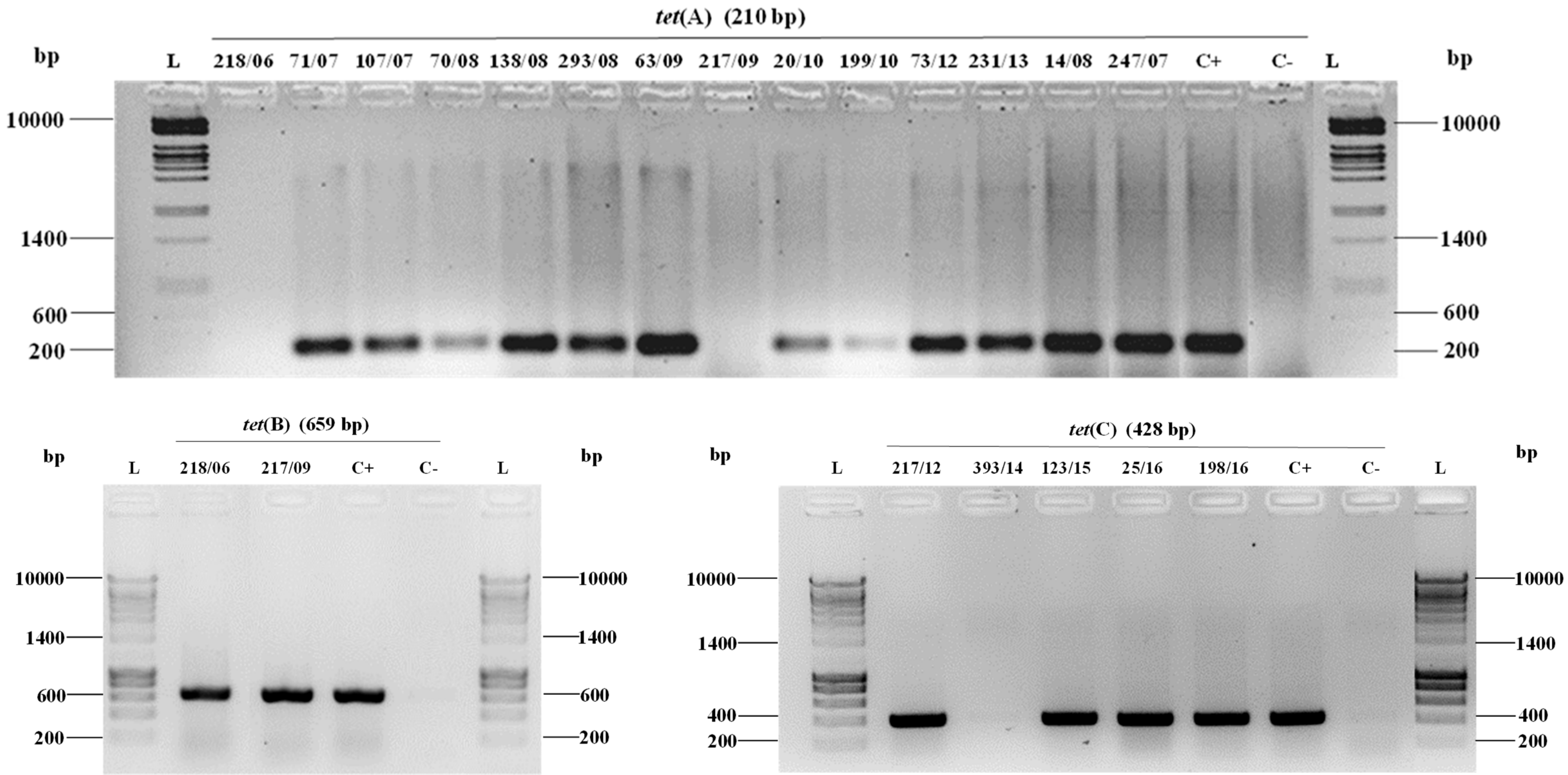

Figure S1. Agarose gels showing examples of PCR fragments amplified with primer pairs specific for the following resistance genes: *fosA7*, *aadA2*, *bla*$_{\text{TEM-1}}$-like, *sul1*, *tet*(A), *tet*(B) and *tet*(C), with the size of the expected amplicons shown in parenthesis. The fragments were visualized in agarose gels (1% in TAE running buffer consisting of 40 mM Tris-acetate pH 8.6, 1 mM EDTA. Lane L, 200 bp ladder (NZYDNA Ladder III, NZYTech, Lisboa, Portugal) used as size marker. Numbers in the other lanes correspond to LSP ("Laboratorio de Salud Pública"; Asturias, Spain) isolates (see Tables 1 and 2 for details); C+, positive control; C-, negative control.

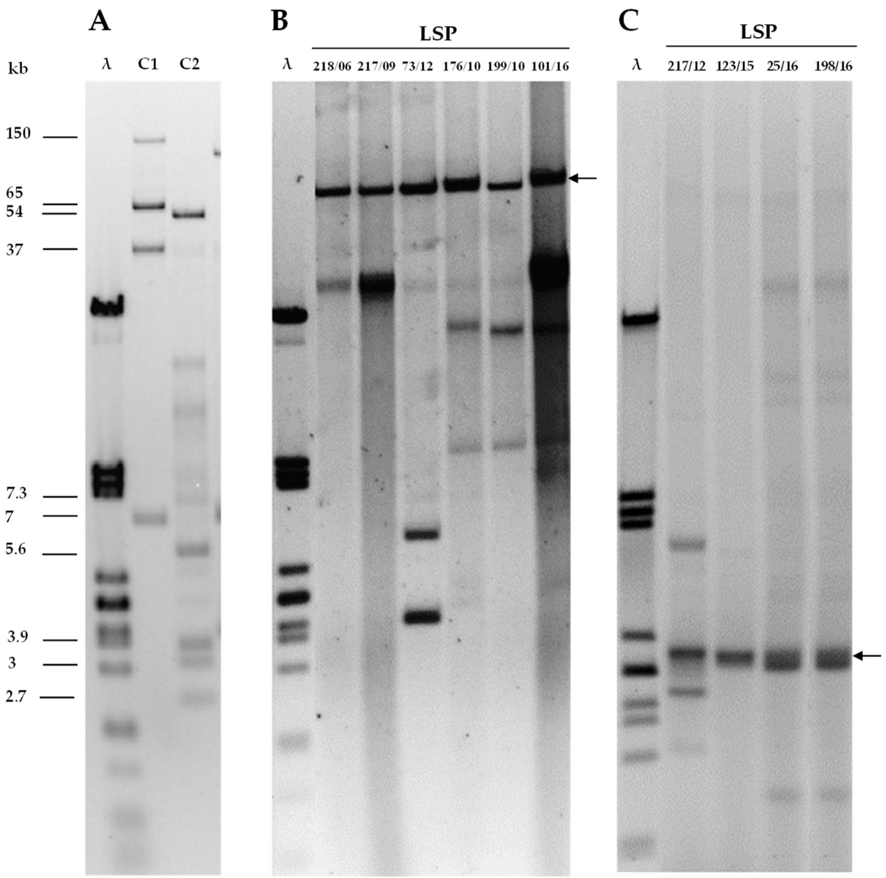

Figure S2. Plasmid profiles of *Salmonella enterica* serovar Derby isolates harboring resistance genes of plasmid location. Plasmids were extracted with the Kado and Liu method [70] and visualized on agarose gels (0.6% in TAE buffer; see legend to Figure S1). **A**. Size standards. Lane λ, lambda phage DNA digested with PstI, included for comparison. Lanes C1 and C2, plasmids extracted from *Escherichia coli* strains 39R861 and V517 [68-70], used as size references for undigested plasmid DNA. **B**. Isolates carrying IncI1-I(α) plasmids (indicated with an arrow). **C**. Isolates carrying pSC101-like plasmids (indicated with an arrow). Other lanes in **B** and **C** correspond to LSP ("Laboratorio de Salud Pública", Asturias, Spain) isolates. Please note that not all plasmids experimentally detected in the isolates were necesarilly identified by PlasmidFinder (see Table 2 for comparison).

References

28. Guerra, B.; Junker, E.; Miko, A.; Helmuth, R.; Mendoza, M.C. Characterization and localization of drug resistance determinants in multidrug-resistant, integron-carrying *Salmonella enterica* serotype Typhimurium strains. *Microb Drug Resist.* 2004, *10,* 83-91. doi: 10.1089/1076629041310136
29. Ng, L. K., Mulvey, M.R., Martin, I., Peters, G.A., Johnson, W. Genetic characterization of antimicrobial resistance in Canadian isolates of *Salmonella* serovar Typhimurium DT104. Antimicrob Agents Chemother. **1999**, 43: 3018-21. doi: 10.1128/AAC.43.12.3018
30. Perreten, V., Boerlin, P. A new sulfonamide resistance gene (*sul3*) in *Escherichia coli* is widespread in the pig population of Switzerland. Antimicrob Agents Chemother. **2003**, 47: 1169-72. doi: 10.1128/AAC.47.3.1169-1172.2003
62. Sandvang, D., Aarestrup, F.M., Jensen, L. B. Characterisation of integrons and antibiotic resistance genes in Danish multiresistant *Salmonella enterica* Typhimurium DT104. *FEMS Microbiol Lett*. **1997**, 157: 177-81. doi: 10.1111/j.1574-6968.1997.tb12770.x
63. Walker, R. A., Lindsay, E., Woodward, M.J., Ward, L.R., Threlfall, E.J. Variation in clonality and antibiotic-resistance genes among multirresistant *Salmonella enterica* serotype Typhimurium phage-type U302 (MR U302) from humans, animals and foods. *Microb Drug Resist*. **2001**, 7:13-21. doi: 10.1089/107662901750152701
64. Madsen, L., Aarestrup F. M., Olsen J.E. Characterisation of streptomycin resistance determinants in Danish isolates of *Salmonella* Typhimurium. *Vet Microbiol*. **2000,** 75: 73-82. doi: 10.1016/s0378-1135(00)00207-8
65. Arlet, G., Phillippon, A. Construction by polymerase chain reaction and intragenic DNA probes for three main types of transferable β-lactamases (TEM, SHV, CARB). *FEMS Microbiol Lett*. **1991**, 82: 19-26. doi:10.1016/0378-1097(91)90414-6
66. Chu, C., Chiu, C. H., Wu, W. Y., Chu, C. H., Liu, T. P., Ou, J.T. Large drug resistance virulence plasmids of clinical isolates of *Salmonella enterica* serovar Choleraesuis. *Antimicrob Agents Chemother*. **2001**, 45: 2299-303. doi: 10.1128/AAC.45.8.2299-2303.2001
67. Ng, L. K., Martin, I., Alfa, M., Mulvey, M. Multiplex PCR for the detection of tetracycline resistant genes. *Mol Cell Probes*. **2001**, 15(4): 209-215. doi: 10.1006/mcpr.2001.0363
68. Kado, C.I., Liu, S.T. Rapid procedure for detection and isolation of large and small plasmids. *J Bacteriol* **1981**, 145, 1365-1373. doi: 10.1128/jb.145.3.1365-1373.1981
69. Threlfall, E. J., Rowe, B., Ferguson, J.l., Ward, L.R. Characterization of plasmids conferring resistance to gentamicin and apramycin in strains of *Salmonella typhimurium* phage type 204c isolated in Britain. *J Hyg (Lond)*. **1986,** 97(3):419-26. doi: 10.1017/s0022172400063609
70. Sánchez, F., Jiménez, G., Aguilar, A., Baquero, F., Rubio, V. Plasmid pVA517C from *Escherichia coli* V517 is required for the expression of an antibiotic microcin. *J Antibiot* (Tokyo). **1986**, 39(7):1028-30. doi: 10.7164/antibiotics.39.1028
